# Innovative DIEP flap perfusion evaluation tool: Qualitative and quantitative analysis of indocyanine green-based fluorescence angiography with the SPY-Q proprietary software

**DOI:** 10.1371/journal.pone.0217698

**Published:** 2019-06-25

**Authors:** Noémie Girard, Myriam Delomenie, Caroline Malhaire, Delphine Sebbag, Aurélie Roulot, Anne Sabaila, Benoît Couturaud, Jean-Guillaume Feron, Fabien Reyal

**Affiliations:** 1 Department of Gynecological and Breast Oncological Surgery, Curie Institute, Paris, France; 2 Department of Radiology, Curie Institute, Paris, France; 3 Department of Plastic and Reconstructive Surgery, Curie Institute, Paris, France; 4 Université de Paris, Université Paris Descartes, Paris, France; di Pompeo d’Illasi, Universita degli Studi di Roma La Sapienza Facolta di Medicina e Psicologia, ITALY

## Abstract

**Background:**

Perfusion-related complications remain the most common concern in DIEP flap breast reconstruction. Indocyanine green-based fluorescence angiography can be used for the real-time intra operative assessment of flap perfusion. The SPY Elite system is the most widely used device in this setting. The main objective was to describe the use of SPY-Q proprietary software to perform qualitative and quantitative analysis of flap perfusion.

**Methods:**

This retrospective cohort study was performed at the Curie Institute between 2013 and 2017. We included patients undergoing unilateral DIEP flap breast reconstruction for whom indocyanine green-based angiography videos were of sufficient quality for analysis. Videos were recorded with the SPY Elite System and analyzed with SPY-Q proprietary software.

**Results:**

We included 40 patients. We used real-time dynamic color analysis to describe three different patterns of flap perfusion. SPY-Q proprietary software provides quantitative flap perfusion parameters. Our quantitative analysis confirmed that zone I is the best perfused part of the flap and zone IV the less perfused one. There was no significant association between flap perfusion pattern and perforator anatomy, patients’ clinical characteristics or postoperative outcomes. After exploratory univariate analysis, quantitative perfusion parameters were significantly impaired in young patients with diabetes mellitus or under hormone therapy by tamoxifen.

**Conclusions:**

We here describe a new approach to assess DIEP flap perfusion using the SPY Elite System proprietary software. It provides interesting qualitative and quantitative analysis that can be used in further studies to precisely assess DIEP flap perfusion.

## Introduction

Deep inferior epigastric perforator (DIEP) flap is currently a popular choice for autologous breast reconstruction. Breast reconstruction from a DIEP flap was first described by Allen *et al*. in 1994 [1]. It provides good esthetic long-term results but requires a trained surgical team. Postoperative complications rate is about 5% [2,3]. The most common locoregional concern remains perfusion-related complications (partial or total necrosis of the flap). That is why accurate pre- and intraoperative assessment of flap perfusion remains a challenge.

Fluorescence angiography, mostly using indocyanine green, is a real-time imaging technique that has already proved useful during breast surgery [4–7]. In procedures involving autologous flaps (transverse rectus abdominis musculocutaneous (TRAM) or DIEP flap), indocyanine green-based angiography appears to be of limited interest for preoperative selection of perforators or for postoperative monitoring. Nonetheless, it seems useful for the intraoperative evaluation of flap perfusion and immediate assessment of the quality of the microvascular anastomosis [8,9]. Indeed, some authors reported that flap areas with poor clinical aspect on intraoperative angiography were those where perfusion-related complications occurred [7,10–13].

The SPY Elite System is the most widely used device for indocyanine green-based angiography in breast reconstructive surgery [13]. The proprietary SPY-Q software (Novadaq, Mississauga, Canada) can perform various analysis of fluorescence angiography data. Several perfusion parameters can be determined and compared between different anatomical regions. Some authors have suggested that SPY-Q software analysis could be used to evaluate the risk of postoperative necrosis after mastectomy with immediate prosthetic breast reconstruction [6,7,14,15].

In this study, we describe how the proprietary SPY-Q software can be used to assess DIEP flap perfusion. Secondarily, we aimed to identify potential factors associated with flap perfusion quantitative parameters.

## Methods

This retrospective cohort study was carried out at the Curie Institute from 2013 to 2017. Formal written approval for research was obtained for each patient treated in our institution. Institutional ethics committee of Curie Institute specifically approved this study.

### Videos selection and data collection

We collected all fluorescence angiography videos recorded during unilateral DIEP flap breast reconstruction procedures during the study period. For this study, we selected videos recorded before the clamping of the epigastric vessels at their origin, after the main perforator had been fully dissected. The SPY Elite System was used for detection. It was placed over the abdominal flap and kept stationary. Each patient received a peripheral intravenous injection of 5 mg Infracyanine (indocyanine green) immediately followed by a peripheral intravenous injection of 10 mL of saline serum. Video recording was initiated at the time of Infracyanine injection and was stopped when the senior surgeon considered fluorescence of the flap to be stable.

We included all patients undergoing unilateral DIEP flap breast reconstruction for whom the video recorded were of sufficient quality for analysis. The quality criteria for video selection were as follows: film duration greater than 20 seconds, shooting centered on the abdominal flap, no camera movements, no additional image in the field of view (e.g. surgical instruments or other tissues), no previous background fluorescence. Videos must also have been recorded with SPY Elite System default recording parameters. For each patient included, clinical data were gathered in the computerized medical file: age, body mass index (BMI), menopausal status, cardiovascular risk factors (hypertension, dyslipidemia, diabetes mellitus, smoking status (divided into “former smoker”, when ancient smoking was stopped less than three years before surgery, or “non-smoker”. Current smoking was a contraindication for DIEP procedure)), treatment by radiotherapy, chemotherapy or hormone therapy (tamoxifen or aromatase inhibitor). Surgical data were searched for in the surgical report validated by the senior surgeon in charge of the patient: time of breast reconstruction, intraoperative surgical difficulties (difficult dissection or wound of the main perforator, immediate refection of anastomosis, intraoperative partial resection of the flap due to abnormal poor tissular perfusion or any difficulties explicitely described in surgery report). Postoperative outcomes (reoperation, prolonged local care, duration of local care) were also gathered in the medical file.

### Preoperative perforators mapping

A thoracoabdominal and pelvic computed tomography (CT) scan with contrast injection was obtained for each patient before surgery. A locoregional or metastatic recurrence of the initial disease was first searched for. The deep inferior epigastric artery and its perforating vessels were then identified and the following characteristics were reported: emergence side, type of bifurcation of the deep inferior epigastric artery (I, II, III [16]), origin of the perforating artery in case of type II or III bifurcation (lateral, intermediate, medial, pararectal medial), main perforating artery diameter (thin < 1mm, intermediate 1–1.5mm, large >1.5mm) [17,18]. The intramuscular course of each perforator was determined by multiplanar reconstruction. The main perforator was defined as the one with the largest arterial caliber and the shortest intramuscular course. The length of intramuscular course was the most important criteria. Recipient vessels were also studied on pre operative CT scan.

### Video analysis using SPY-Q software

Video recordings were analyzed using proprietary SPY-Q software. Authors verified that initial recording parameters were similar among selected videos (especially ambient light correction). First, all videos were cut 20 seconds after the beginning of the recording to have all videos the same length and enable comparisons.

**Fig 1:**
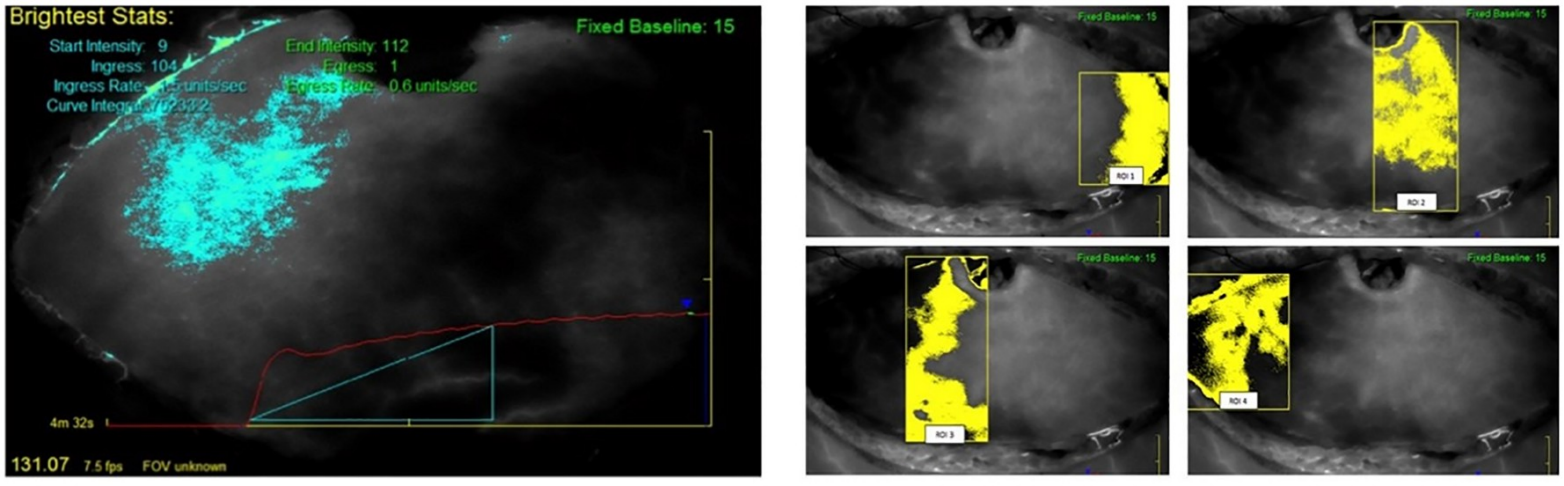
Flap perfusion analyzed with the “Autoview” function. Fig 1A: the bright zone on the left is the flap zone where fluorescence is the higher and on which perfusion parameters are estimated. The red curve is the curve of fluorescence across the time. Fig 1B: Definition of the four regions of interest (ROI). Yellow-colored zones are used to calculate perfusion parameters.

On each video, a dynamic color analysis was first performed with the "Overview" function. After that, a quantitative analysis was performed, first on the whole flap, and then, on four regions of interest. Ingress and ingress rate were the two quantitative perfusion parameters selected for the analysis of flap perfusion. Ingress (in absolute perfusion units, APU) is the absolute difference between initial mean fluorescence in a region of interest and its maximal value. Ingress rate (in absolute perfusion units per second, APU/s) is the fluorescence blush rate in a region. Whole flap perfusion was first analyzed with the "Autoview" function (Fig 1A). Then, four regions of interest (ROI) were defined with the "Region" function of the software as follows: ROI 1 was the peripheral flap end ipsilateral to the emergence of the main perforator (commonly described as Hartrampf Zone III [19]); ROI 2 was the main perforator emergence area (Hartrampf Zone I); ROI 3 was the flap area adjacent to the main perforator emergence area across the midline (Hartrampf Zone II); and ROI 4 was the peripheral flap end contralateral to the area of emergence of the main perforator (Hartrampf Zone IV) (Fig 1B).

### Statistical analysis

Categorical covariates were described by raw numbers and associated percentages for each category. Quantitative covariates were summarized by their mean and standard deviation or their median and extreme values. Categorical covariates were compared in χ^2^ test or Fisher’s exact tests, according to conditions of validity of the tests. Means of continuous covariates were compared in Student’s t test or by analysis of variance. Differences in means or percentages were considered significant if a p value below 0.05 was obtained. Multivariate analysis was not performed because of the limit statistical validity and the exploratory design of the study. All statistical analysis were performed with R software (R Development Core Team, 2009), version 3.1.2.

## Results

### Study population

From 2013 to 2017, 47 indocyanine green-based angiography videos were recorded for flap perfusion assessment after the dissection of the main perforator during unilateral DIEP flap breast reconstructions. All flaps were one-perforator flaps. Of these, 40 were retained for analysis. Others were discarded because of insufficient quality of the recording.

We thus included 40 patients. Mean age was 51 years old (y-o) (range 30 to 71 y-o). Twenty-one patients (53%) were overweight and 24 (60%) had at least one cardiovascular risk factor. All mastectomies were performed in breast cancer treatment setting. The breast reconstruction procedure was secondary in thirty patients (75%). Twenty-four patients (60%) were on adjuvant hormone therapy.

### Postoperative outcomes

Five patients (13%) underwent repeated surgery for flap-related complications: venous thrombosis (n = 2), arterial thrombosis (n = 1), haemorrhage (n = 1) and partial flap necrosis (n = 1). Two flaps were removed (venous thrombosis on the first postoperative day (n = 1) and partial flap necrosis with delayed local healing (n = 1)).

Postoperative healing care was prolonged for 10 patients, due to abdominal scar in half these cases. The patients’ characteristics and surgical outcomes are summarized in Table 1.

**Table 1:** Study population.

Clinical characteristic	n = 40
Age (median, min-max)	53 (42–59)
Body mass index (kg/m^2^) (median, min-max)	25.3 (22.3–28.1)
Overweight (BMI > 25 kg/m^2^)	21 (52.5%)
Hypertension	6 (14.6%)
Dyslipidemia	4 (10%)
Diabetes mellitus	2 (5%)
Smoking stopped < 3 years	5 (12.5%)
At least one cardiovascular risk factor	24 (60%)
Breast cancer treatment	
Hormone therapy	24 (60%)
Tamoxifen	19
Aromatase inhibitor	5
Chemotherapy	27 (68%)
Radiotherapy	27 (68%)
Surgery	
Secondary reconstruction	30 (75%)
Surgical difficulties	4 (10%)
Reoperation	5 (12.5%)
Venous thrombosis	2
Arterial thrombosis	1
Flap necrosis	1
Hemorrhage	1
Prolonged local care	10 (25%)
Duration of local care (days, median, min-max)	61 (21–150)

### Flap perfusion

**Fig 2:**
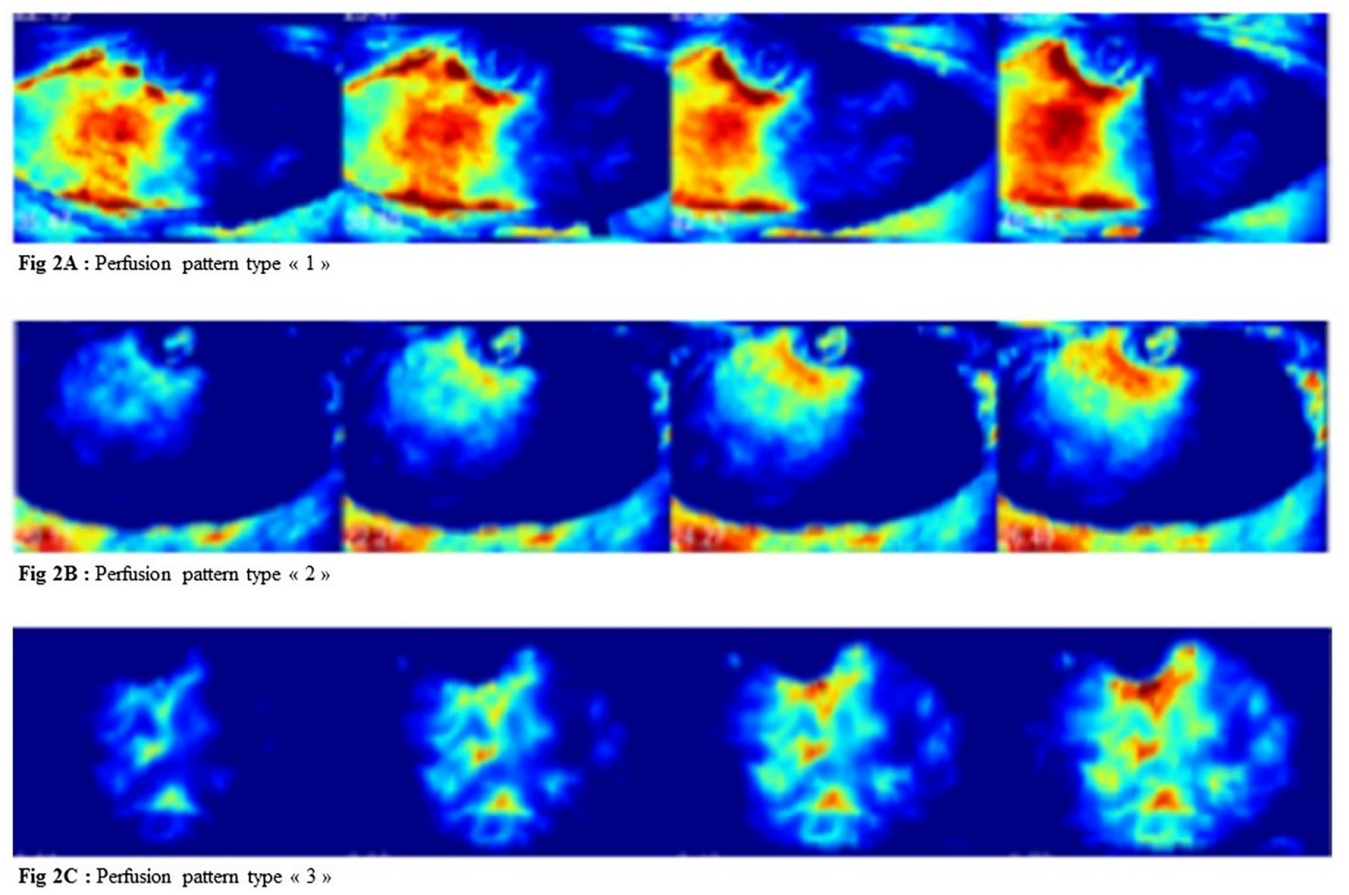
Flap perfusion patterns. Fig 2A: Perfusion pattern type «1». Fig 2B: Perfusion pattern type «2». Fig 2C: Perfusion pattern type «3». Dynamic color analysis representing fluorescence of the flap over time. Red zone is the best perfused one and blue one, the less perfused one.

We identified three distinct flap perfusion patterns using color analysis, defined as follows: type 1 pattern (n = 14): homogeneous flap perfusion ipsilateral to the main perforator emergence without crossing the midline (Fig 2A); type 2 pattern (n = 16): limited flap perfusion with good perfusion around the area of perforator emergence and poor perfusion of the rest of the flap (Fig 2B); type 3 pattern (n = 10): homogeneous perfusion on either side of the midline and poor perfusion of peripheral flap ends (Fig 2C).

In whole flap perfusion quantitative analysis, median ingress was 127 APU (interquartile range (IQR) = [86–167]) and median ingress rate was 9.4 APU/s (IQR = [4.7–14.4]).

**Fig 3:**
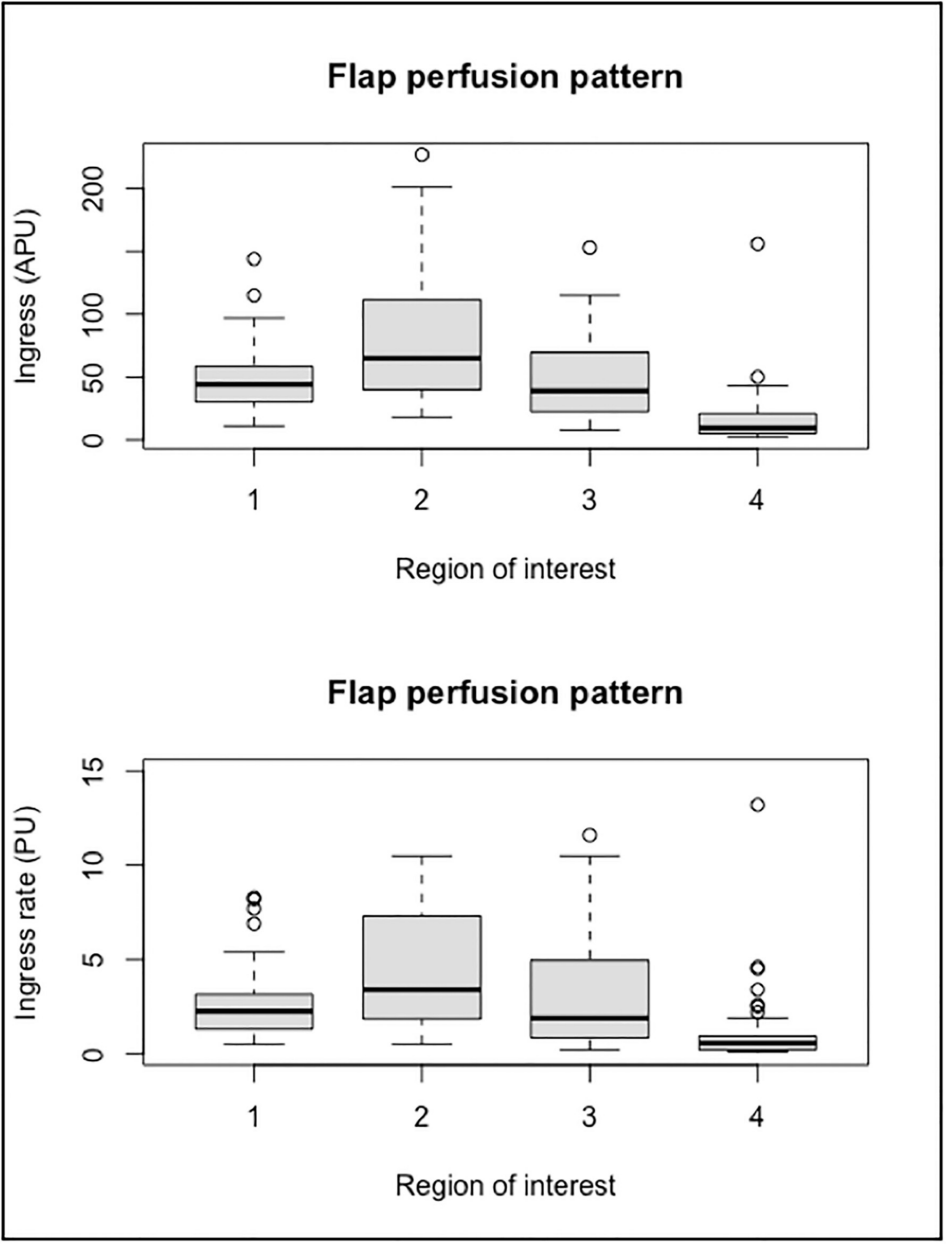
Flap zones and quantitative perfusion parameters. This boxplot figure represents distributions of ingress (above) and ingress rate (below) calculated on the four flap zones as defined above. When quantitatively assessed, perfusion is the higher in ROI 2 (zone I) and the lower in ROI 4 (zone IV).

The flap region with the best perfusion was the main perforator emergence area (zone I): median ingress was 65 APU (IQR = [41–110]) and median ingress rate was 3.4 APU/s (IQR = [1.9–7.1]). These perfusions parameters were significantly higher than those of the other zones of the flap (*p <0*.*01*). The peripheral flap end contralateral to the area of emergence of the main perforator (zone IV) had the lowest perfusion parameters of all zones. No difference was observed between the flap zones located on either side of the region of emergence of the main perforator (zone II and III) (Fig 3).

### Flap perfusion and perforators anatomy

**Fig 4:**
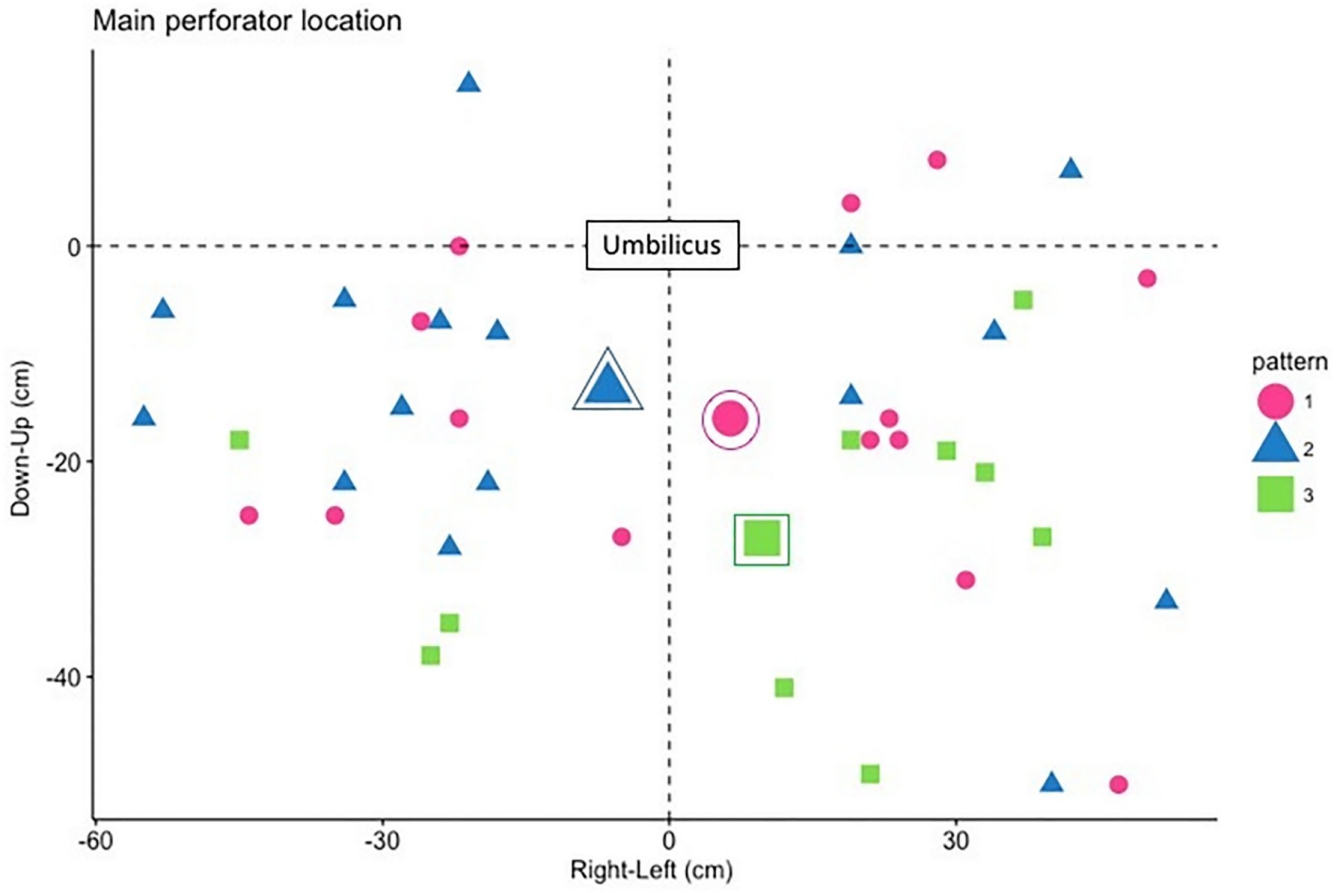
Flap perfusion pattern and main perforator location. Circled symbol represents the mean location of perforator emergence for each perfusion pattern (as defined above).

The main perforators (determined on the preoperative CT scan) were mostly medial row perforators (n = 21 (53%)) with a large diameter (> 1.5mm, n = 29 (73%)) and type II bifurcation was the most common (n = 33 (83%)). There was no significant association between the anatomical characteristics of the perforators (diameter, bifurcation type and origin) and perfusion patterns as defined above. In the type 3 pattern, the main perforator emerged lower on the flap than in the type 1 and 2 patterns (Fig 4). There was no significant association between the anatomical characteristics of the perforators (diameter, bifurcation type and origin) and quantitative flap perfusion parameters, over the entire flap or in any particular zone.

The results of univariate analysis of association between the main perforator anatomical characteristics and the flap perfusion parameters are presented in Table 2.

**Table 2:** Univariate analysis between flap perfusion parameters and anatomical characteristics of the main perforator.

		Flap perfusion pattern			Whole-flap perfusion				Hartrampf zone I perfusion			
	n	1 (n = 14)	2 (n = 16)	3 (n = 10)	Ingress (APU)	*p value*	Ingress rate (APU/s)	*p value*	Ingress (APU)	*p value*	Ingress rate (APU/s)	*p value*
**Diameter**						*0*.*89*		*0*.*59*		*0*.*77*		*0*.*93*
**Thin (<1 mm)**	1 (2%)	1 (7%)	0	0	127		17.1		95		6.1	
**Medium (1–1.5 mm)**	10 (25%)	5 (36%)	3 (19%)	2 (20%)	136		11.5		95		7.04	
**Large (>1.5 mm)**	29 (73%)	8 (57%)	13 (81%)	8 (80%)	126		9.85		80		5.95	
**Bifurcation type**						*0*.*49*		*0*.*62*		*0*.*35*		*0*.*63*
**I**	6 (15%)	1 (7%)	4 (25%)	1 (10%)	104		8.23		53.5		3.38	
**II**	33 (83%)	13 (93%)	12 (75%)	8 (80%)	132		10.8		89.2		6.72	
**III**	1 (2%)	0	0	1 (10%)	152		10.2		97		6.9	
**Origin if type II/III**						*0*.*85*		*0*.*75*		*0*.*99*		*0*.*89*
**Intermediate**	1 (2%)	0	0	1 (11%)	152		10.2		97		6.9	
**Lateral**	5 (13%)	3 (23%)	2 (17%)	0	128		12.2		85		6.86	
**Medial**	21 (53%)	10 (77%)	6 (50%)	5 (56%)	132		9.68		90.7		5.96	
**Medial pararectal**	7 (32%)	0	4 (33%)	3 (33%)	148		13.4		87.9		8.90	

### Flap perfusion and clinical features

The type of flap perfusion pattern was not significantly associated with patients’ clinical characteristics or clinical postoperative outcomes (results not shown).

**Fig 5:**
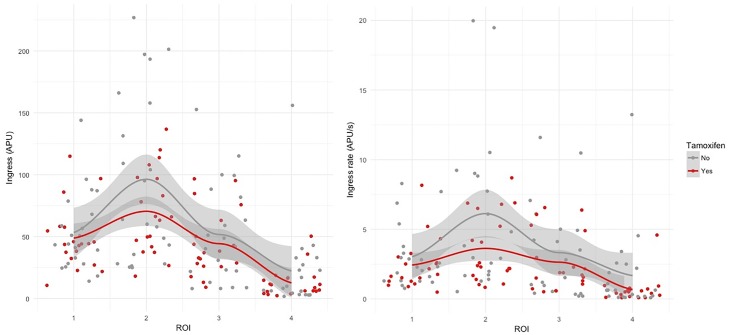
Flap perfusion and tamoxifen. Fig 5A: Flap perfusion assessed by ingress and tamoxifen. Fig 5B: Flap perfusion assessed by ingress rate and tamoxifen.

After univariate analysis, perfusion of the ROI 2 (zone I) was significantly better in patients over the age of 60 years than in other patients (mean ingress 130 APU versus 68.8 APU, *p = 0*.*04* and mean ingress rate of 13.7 APU/s versus 3.74 APU/s, *p = 0*.*03*). Whole flap perfusion, whether assessed by ingress or ingress rate, was significantly impaired in patients with diabetes mellitus on univariate analysis (respectively, mean ingress = 45.5 APU versus 133 APU, *p <0*.*001* and mean ingress rate = 2.20 APU/s versus 10.9 APU/s, respectively, *p = 0*.*002*). Flap perfusion was not significantly associated with other cardiovascular risk factors. Flap perfusion assessed by ingress rate was impaired in patients on tamoxifen-based hormone therapy after univariate analysis. Indeed, mean ingress rate in the ROI 2 (zone I) was significantly lower in patients under tamoxifen than in patients without hormone therapy or on aromatase inhibitor treatment (3.65 APU/s versus 8.49 APU/s, *p = 0*.*04*) (Fig 5A and 5B).

The results of the univariate analysis between flap perfusion parameters and patients’ clinical characteristics are presented in Table 3.

**Table 3:** Univariate analysis of the relationship between flap perfusion parameters and patients’ clinical characteristics.

	Ingress (APU)	*p*	Ingress rate (APU/s)	*p*	Ingress zone I (APU)	*p*	Ingress Rate zone I (APU/s)	*p*
**Cardiovascular risk factors**								
Age								
< 60 years	116	*0*.*07*	8.74	*0*.*09*	68.8	*0*.04*	3.74	*0*.03*
> 60 years	165		15.5		130		13.7	
Overweight								
No	133	*0*.*63*	10.9	*0*.*71*	86.9	*0*.*74*	5.16	*0*.*40*
Yes	124		10.0		86.6		7.19	
Smoking status								
Never	128	*0*.*80*	10.4	*0*.*97*	80.4	*0*.*30*	6.43	*0*.*40*
Ancient, stopped > 3 years	134		10.5		110		4.80	
Hypertension								
No	124	*0*.*41*	9.23	*0*.*19*	75.4	*0*.*14*	4.26	*0*.*11*
Yes	156		17.3		133		17.4	
Dyslipidemia								
No	125	*0*.*44*	9.49	*0*.*32*	79.5	*0*.*37*	4.97	*0*.*25*
Yes	162		19.0		125		17.5	
Diabetes mellitus								
No	133	*<0*.001*	10.9	*0*.002*	86.5	*0*.02*	6.32	*0*.*23*
Yes	45.5		2.20		37.5		4.50	
**Cancer treatment**								
Hormone therapy								
None or aromatase inhibitors	141	*0*.*12*	11.7	*0*.*25*	96.3	*0*.*14*	8.57	*0*.04*
Tamoxifen	114		9.00		70.5		3.63	
Radiotherapy								
No	128	*0*.*96*	11.4	*0*.*66*	93.8	*0*.*53*	8.92	*0*.*23*
Yes	129		10.0		79.4		4.93	
Chemotherapy								
No	127	*0*.*93*	11.0	*0*.*81*	92.1	*0*.*61*	8.47	*0*.*33*
Yes	129		10.2		80.2		5.14	

## Discussion

### A new accurate and objective tool to assess DIEP flap perfusion

The main objective of this exploratory study was to describe the use of SPY-Q proprietary software for DIEP flap perfusion assessment. To date, this is the first study to describe the type of analysis that can be performed on indocyanine green based-angiography using SPY device in DIEP flap procedure: 1/color intra operative dynamic analysis, 2/quantitative whole flap or pre-defined region of interest perfusion analysis. This is the largest serie of patients reported with SPY scanning of DIEP flaps in breast reconstruction. It is also the first report of the use of SPY-Q proprietary software in this setting. We here propose a new approach for DIEP flap perfusion assessment. We show that flap perfusion can be both qualitatively and quantitatively evaluated quite easily.

Indeed, real-time color analysis can facilitate indocyanine green-based angiography interpretation and be useful for intra operative flap harvest and design. Precise quantitative analysis of flap perfusion is possible with SPY-Q software. Although it seems of little interest in intra operative setting or pre operative perforator mapping, we suggest that it could be an interesting tool to use when studying DIEP flap perfusion. In many reported studies, DIEP flap perfusion is generally clinically assessed. We think that quantitative analysis with SPY-Q proprietary software, using parameters such as ingress and ingress rate, could refine and precise the results.

Our DIEP flap perfusion analysis using selected quantitative parameters (ingress and ingress rate) confirm that zone I is the best perfused zone and that zone IV is the less perfused one. Our results seem consistent with those previously reported in literature and our quantitative analysis seems then accurate for DIEP flap perfusion assessment. Lee *et al*. recently published a meta-analysis of relevant clinical and anatomical DIEP flap perfusion studies [20]. It is universally held that Hartrampf zone I is the best perfused tissue and Hartrampf zone IV, the less perfused one. But there are discrepancies between the results of *ex vivo* and clinical studies regarding perfusion of Hartrampf zone II and zone III. Contrarily to *ex vivo* studies, in clinical studies, perfusion of these two zones does not seem to depend on the type of perforator (medial or lateral) and zone III seems to be systematically better perfused than zone II. We did not find any differences between zones II and III perfusion. That can be explained by a lack of power due to the small number of patients.

Another part of the analysis confirms that quantitative analysis with SPY-Q software is an accurate objective assessment of clinical flap perfusion. Dynamic color analysis showed three patterns of flap perfusion: the type 1 pattern, in which perfusion is homogeneous but limited in the hemi-abdomen in which the main perforator emerges; the type 2 pattern, in which perfusion is good around the emergence of the main perforator and poor elsewhere in the flap; and the type 3 pattern, in which flap perfusion is homogeneous across the midline. In type 3 pattern, the perforator emergence seems generally lower than in other patterns. The results of the quantitative analysis were consistent with those of the color analysis, which means that ingress and ingress rate are well related to clinical assessment of flap perfusion.

### Predictive and protective factors for flap perfusion

A secondary objective of our study was to explore potential risk factors for altered quantitative perfusion parameters. From univariate analysis, age < 60 y.o, diabetes mellitus and tamoxifen-based hormone therapy were statistically related to lower perfusion values. Because it was an exploratory study, we chose not to perform multivariate analysis, which can limit the validity of conclusions from this analysis. Moreover, we recognize that this study did not take into account some factors that can have significant effects on perfusion such as operating room environmental variables (ambient light, core temperature), patients’ variables (core temperature, intra operative and postoperative blood pressure, parity) or anesthesic settings (use of vasoconstrictors, type of intravenous fluid infusion) [21]. For some factors, such as anesthesia that follows a standardized protocole or ambient light that was systematically corrected during software analysis, we think that this leads to only limited bias. Nonetheless, we assume that conclusions can not be drawn from this part of the analysis, since studying association between quantitative perfusion parameters, predictive and protective factors and perfusion-related complications was not the main objective of our exploratory study.

### Perforator anatomy and flap perfusion

Wong *et al*. reported that the main perforator anatomy is related to flap perfusion zones and could be used to adapt surgical indications to the needs of reconstruction [22]. In our population, medial row perforators of pararectal origin seemed to be associated with the best flap perfusion (assessed by quantitative parameters), although statistical significance was not reached. In anatomical studies, medial row perforators are described as having a greater perfusion territory than lateral ones, crossing the midline and extending to the four zones [20]. Moreover, the fact that the perimuscular path is much easier to follow during medial row perforator dissection and the fact that lateral perforator dissection expose to higher risk of nerve damage, suggest the preferential use of medial row perforators in unilateral DIEP flap breast reconstruction.

### Improve DIEP flap reconstruction

Although the DIEP flap is one of the current most popular choice for autologous breast reconstruction, it is still a long, heavy surgical procedure for the patient and perfusion-related complications is the most common concern. Consequently, the main challenges remain 1/ to perform a precise pre operative perforator mapping and choose the best flap harvesting procedure considering perforators’ anatomy and needs for reconstruction, 2/ to precisely evaluate flap perfusion in intra operative setting, 3/ to define protective and predictive factors for perfusion-related complications. Numerous studies focus on how to improve DIEP flap breast reconstruction results and technique. Reported studies have often focused on complications, costs or patients’ satisfaction [23–25]. An original recent study has proposed solutions to reduce DIEP operative time to make it more acceptable for patients [26]. Some authors have also tried to correlate the diameter of the vein to the flap weight in order to help surgeon in the choice of microvascular anastomosis technique [27]. Flap perfusion is still subject to discussions. We here propose a simple way to better clinically assess flap perfusion intra operatively (color analysis) and a tool for objective quantitative analysis of flap perfusion for further studies.

### Conclusion

SPY-Q software provides a precise qualitative and quantitative analysis of flap perfusion during DIEP flap breast reconstruction. Color intra operative analysis can help decision on flap harvesting and design. Moreover, quantitative and objective perfusion parameters seem accurate for flap perfusion assessment and could easily be used in further studies to refine the results. Studies on larger numbers of patients are required to determine the association between quantitative perfusion parameters, perforator anatomy, flap perfusion pattern and predictive factors for perfusion-related complications.

## Supporting information

S1 Data The dataset on which this article is based. (CSV)

## References

[1] Allen RJ, Treece P. Deep inferior epigastric perforator flap for breast reconstruction. Ann Plast Surg. 1994 1;32(1):32–8. 8141534 10.1097/00000637-199401000-00007

[2] Nahabedian MY, Tsangaris T, Momen B. Breast reconstruction with the DIEP flap or the muscle-sparing (MS-2) free TRAM flap: is there a difference? Plast Reconstr Surg. 2005 2;115(2):436–444-446. 15692347 10.1097/01.prs.0000149404.57087.8e

[3] Scheer AS, Novak CB, Neligan PC, Lipa JE. Complications associated with breast reconstruction using a perforator flap compared with a free TRAM flap. Ann Plast Surg. 2006 4;56(4):355–8. 10.1097/01.sap.0000201549.83738.42 16557060

[4] Murray JD, Jones GE, Elwood ET, Whitty LA, Garcia C. Fluorescent intraoperative tissue angiography with indocyanine green: evaluation of nipple-areola vascularity during breast reduction surgery. Plast Reconstr Surg. 2010 7;126(1):33e–34e. 10.1097/PRS.0b013e3181dab2c2 20595841

[5] Brunworth LS, Samson MC, Newman MI, Ramirez JR. Nipple-areola complex evaluation in long pedicled breast reductions with real-time fluorescent videoangiography. Plast Reconstr Surg. 2011 8;128(2):585–586-587. 10.1097/PRS.0b013e31821e71f6 21788855

[6] Munabi NCO, Olorunnipa OB, Goltsman D, Rohde CH, Ascherman JA. The ability of intra-operative perfusion mapping with laser-assisted indocyanine green angiography to predict mastectomy flap necrosis in breast reconstruction: a prospective trial. J Plast Reconstr Aesthetic Surg JPRAS. 2014 4;67(4):449–55. 10.1016/j.bjps.2013.12.040 24507962

[7] Newman MI, Jack MC, Samson MC. SPY-Q analysis toolkit values potentially predict mastectomy flap necrosis. Ann Plast Surg. 2013 5;70(5):595–8. 10.1097/SAP.0b013e3182650b4e 23542838

[8] Pestana IA, Crantford JC, Zenn MR. Correlation between Abdominal Perforator Vessels Identified with Preoperative Computed Tomography Angiography and Intraoperative Fluorescent Angiography in the Microsurgical Breast Reconstruction Patient. J Reconstr Microsurg. 2014 5 6; 10.1055/s-0034-1372478 24801668

[9] Mothes H, Dinkelaker T, Dönicke T, Friedel R, Hofmann GO, Bach O. Outcome prediction in microsurgery by quantitative evaluation of perfusion using ICG fluorescence angiography. J Hand Surg Eur Vol. 2009 4;34(2):238–46. 10.1177/1753193408090399 19369300

[10] Wu C, Kim S, Halvorson EG. Laser-assisted indocyanine green angiography: a critical appraisal. Ann Plast Surg. 2013 5;70(5):613–9. 10.1097/SAP.0b013e31827565f3 23579465

[11] Komorowska-Timek E, Gurtner GC. Intraoperative perfusion mapping with laser-assisted indocyanine green imaging can predict and prevent complications in immediate breast reconstruction. Plast Reconstr Surg. 2010 4;125(4):1065–73. 10.1097/PRS.0b013e3181d17f80 20335859

[12] Duggal CS, Madni T, Losken A. An outcome analysis of intraoperative angiography for postmastectomy breast reconstruction. Aesthet Surg J. 2014 1 1;34(1):61–5. 10.1177/1090820X13514995 24396073

[13] Griffiths M, Chae MP, Rozen WM. Indocyanine green-based fluorescent angiography in breast reconstruction. Gland Surg. 2016 4;5(2):133–49. 10.3978/j.issn.2227-684X.2016.02.01 27047782 PMC4791345

[14] Moyer HR, Losken A. Predicting mastectomy skin flap necrosis with indocyanine green angiography: the gray area defined. Plast Reconstr Surg. 2012 5;129(5):1043–8. 10.1097/PRS.0b013e31824a2b02 22544087

[15] Phillips BT, Lanier ST, Conkling N, Wang ED, Dagum AB, Ganz JC, et al Intraoperative perfusion techniques can accurately predict mastectomy skin flap necrosis in breast reconstruction: results of a prospective trial. Plast Reconstr Surg. 2012 5;129(5):778e–88e. 10.1097/PRS.0b013e31824a2ae8 22544108

[16] Moon HK, Taylor GI. The vascular anatomy of rectus abdominis musculocutaneous flaps based on the deep superior epigastric system. Plast Reconstr Surg. 1988 11;82(5):815–32. 2971981 10.1097/00006534-198811000-00014

[17] Grover R, Nelson JA, Fischer JP, Kovach SJ, Serletti JM, Wu LC. The Impact of Perforator Number on Deep Inferior Epigastric Perforator Flap Breast Reconstruction. Arch Plast Surg. 2014 1;41(1):63–70. 10.5999/aps.2014.41.1.63 24511497 PMC3915159

[18] Lam DL, Mitsumori LM, Neligan PC, Warren BH, Shuman WP, Dubinsky TJ. Pre-operative CT angiography and three-dimensional image post processing for deep inferior epigastric perforator flap breast reconstructive surgery. Br J Radiol. 2012 12;85(1020):e1293–7. 10.1259/bjr/30590223 23175495 PMC3611736

[19] Hartrampf CR, Scheflan M, Black PW. Breast reconstruction with a transverse abdominal island flap. Plast Reconstr Surg. 1982 2;69(2):216–25. 6459602 10.1097/00006534-198202000-00006

[20] Lee K-T, Mun G-H. Perfusion of the diep flaps: A systematic review with meta-analysis. Microsurgery. 2018 1;38(1):98–108. 10.1002/micr.30024 26773334

[21] Santanelli F, Longo B, Cagli B, Pugliese P, Sorotos M, Paolini G. Predictive and protective factors for partial necrosis in DIEP flap breast reconstruction: does nulliparity bias flap viability? Ann Plast Surg. 2015 1;74(1):47–51. 10.1097/SAP.0b013e31828d994d 23851375

[22] Wong C, Saint-Cyr M, Mojallal A, Schaub T, Bailey SH, Myers S, et al Perforasomes of the DIEP flap: vascular anatomy of the lateral versus medial row perforators and clinical implications. Plast Reconstr Surg. 2010 3;125(3):772–82. 10.1097/PRS.0b013e3181cb63e0 20195105

[23] Zhong T, Novak CB, Bagher S, Maass SWMC, Zhang J, Arad U, et al Using propensity score analysis to compare major complications between DIEP and free muscle-sparing TRAM flap breast reconstructions. Plast Reconstr Surg. 2014 4;133(4):774–82. 10.1097/PRS.0000000000000024 24675183

[24] Fischer JP, Sieber B, Nelson JA, Cleveland E, Kovach SJ, Wu LC, et al Comprehensive outcome and cost analysis of free tissue transfer for breast reconstruction: an experience with 1303 flaps. Plast Reconstr Surg. 2013 2;131(2):195–203. 10.1097/PRS.0b013e318277856f 23357982

[25] Chang EI, Chang EI, Soto-Miranda MA, Zhang H, Nosrati N, Crosby MA, et al Comprehensive Evaluation of Risk Factors and Management of Impending Flap Loss in 2138 Breast Free Flaps. Ann Plast Surg. 2016 1;77(1):67–71. 10.1097/SAP.0000000000000263 25003429

[26] Laporta R, Longo B, Sorotos M, Farcomeni A, Amorosi V, Santanelli di Pompeo F. Time-dependent factors in DIEP flap breast reconstruction. Microsurgery. 2017 10;37(7):793–9. 10.1002/micr.30203 28758229

[27] Rubino C, Ramakrishnan V, Figus A, Bulla A, Coscia V, Cavazzuti MA. Flap size/flow rate relationship in perforator flaps and its importance in DIEAP flap drainage. J Plast Reconstr Aesthetic Surg JPRAS. 2009 12;62(12):1666–70. 10.1016/j.bjps.2008.05.045 18851934

